# High expression of CD34 and α6-integrin contributes to the cancer-initiating cell behaviour in ultraviolet-induced mouse skin squamous cell carcinoma

**DOI:** 10.7150/jca.45819

**Published:** 2020-09-30

**Authors:** Hengning Ke, YvYing Yang, Yuan Lin, Li Liu, Jianmin Sun, Ramin Massoumi

**Affiliations:** 1 Hubei AIDS Clinical Training Center, Department of Infectious Disease, Zhongnan Hospital, Wuhan University, Wuhan, P.R. China.; 2 Department of Laboratory Medicine, Translational Cancer Research, Lund University, Medicon Village, Lund, Sweden.; 3 Cancer Research Institute, General Hospital, Ningxia Medical University, Yinchuan, P.R. China.; 4 School of Basic Medical Sciences, Ningxia Medical University, Yinchuan, P.R. China.

**Keywords:** UV-B, CD34, α6-integrin, squamous cell carcinoma

## Abstract

Squamous cell carcinoma caused by ultraviolet light exposure represents over 40% of all malignant diseases. It is one of the most commonly found human tumours. Tumour mass within squamous cell carcinoma consists of various cell types, including cancer-initiating cells that are responsible for tumour progression, metastasis and chemoresistance and implicated in clinical relapse. In the present study, we aimed to characterise whether the cell population with high CD34 and α6-integrin expression behave as cancer-initiating cells within ultraviolet-induced squamous cell carcinoma in mouse skin. CD34^high^α6-integrin^high^ compared to CD34^low^α6-integrin^high^ cells isolated from ultraviolet-induced squamous cell carcinoma could propagate effectively by displaying greater tumour initiating and self-renewal abilities. Our study suggests that CD34^high^α6-integrin^high^ cells act as initiators upon ultraviolet-induced skin squamous cell carcinoma.

## Introduction

The incidence rate of skin tumours for both non-melanoma and melanoma is rapidly increasing. Solar radiation, as well as long-term exposure to ultraviolet (UV-B) radiation, has the greatest impact on development of these skin tumours 1. Non-melanoma skin tumours, including basal cell carcinoma (BCC) and squamous cell carcinoma (SCC), usually develop on chronically photo-exposed skin areas. Patients who suffer from SCC metastasis have a poor prognosis due to the low survival rate over 10 years 2-4. SCC can metastasise to distant organs and is usually refractory to conventional therapy 5. Upon UV-B radiation, cyclobutane pyrimidine dimers (CPDs) form within cells and cause C-T or CC-TT replacements that result in signature p53 mutations 6, 7. The DNA damage could also result from reactive oxygen species produced during UV-B radiation. The oncogenic processes of UV treatment involve immune suppression, inactivation of tumour suppressor p53 and/or phosphatase and tensin homolog (Pten), and activation of Ras oncogene 8. However, the underlying cellular mechanisms that drive the UV-induced tumour progression are not well known. Here, we used a mouse model with UV-radiated skin tumours, which resemble malignant human SCC and provided an accessible model to mimic the processes of human tumour initiation and growth.

Cancer-initiating cells are believed to be the driving force for cancer progression, metastasis and relapse after therapy 9. The development of therapeutic strategies that target cancer-initiating cells depends on the characterisation of cell surface markers. Within the tumour tissues, various cell populations with different gene expression profiles can be identified. Among these cells, the long-lived epidermal cancer-initiating cells may be the origin of SCC under tumourigenic events such as human papilloma virus (HPV) infection, UV-radiation and/or chemical carcinogen treatments 10. Bulge stem cells and other epidermal lineages can induce papilloma or malignant tumours depending on various genetic hits 11. Even though there are multiple identified cancer-initiating markers, these parameters can differ among cancer types. Most of the identified cell surface markers suggest that cancer-initiating cells originate from embryonic or adult stem cells via the accumulation of epigenetic and genetic alterations 12. Recently, both Lgr5 and Lgr6 were reported to be epithelial stem cell markers in SCC 13, 14.

Cancer-initiating cells can be separated and sorted by cell surface expression markers. The cell surface glycoprotein CD34 is most commonly used for mouse bulge stem cell isolation 15. Previous studies demonstrated the expression of CD34 in haematologic progenitor 16 and hair follicle progenitor cells 17. Furthermore, cancer cells that express high or low CD34 levels play a significant role in mouse models with genetic hits or chemical carcinogens 18, 19. Additionally, CD34-sorted cells elevate the tumour initiating ability of cancer cells compared to non-sorted cells 20. However, the role of cancer cells with high CD34 expression in UV-induced skin cancer is unknown. In addition to CD34, α6-integrin (CD49f) has also been identified in stem cell populations of keratinocytes 21. α6-integrin is also expressed in cancer stem cells (including SCC stem cells) 19. *In vitro*, α6-integrin-positive cancer stem cells exhibit enhanced tumourigenic capabilities and resistance to radiation therapies 22, 23. Cancer-initiating cells derived from established cervical cancer cell lines exhibit stem cell markers and increased radioresistance 23. In glioblastoma cancer stem cells, cells with high α6-integrin expression, form tumourspheres *in vitro* and propagate to form tumours in transplantation assays *in vivo* faster than cells with low α6-integrin expression 24.

Whether CD34 and α6-integrin expression can be considered a cancer stem cells marker in UV-induced skin cancer has not yet been investigated. In the present study, we show that high expression of CD34 and α6-integrin within UV-induced mouse SCC recapitulated cancer-initiating cells.

## Materials and Methods

### Mice and UV irradiation

To establish a UV-induced SCC model, 28 FVB female mice aged 6-8 weeks were housed in a standard animal facility at the university. All animal experiments were conducted according to the guidelines for animal experimentation at Wuhan University. This project was approved by the Ethic Committee of Zhongnan Hospital of Wuhan University (IACUCU2019022). All animals had their backs shaved with electric clippers, placed in the pre-designed cage with the UV-lamp installed above, and irradiated on the back with a 180 mJ/cm^2^ UV dosage for 20 min each day for the first week. Each mouse was kept in a position that only the dorsal shaved surface was exposed to UV-light and tumour was appearing only in this site. The lamps used in the study were TL 40W/01 RS UVB-Narrowband from Phillips and the predominant wavelength was between 300 and 315 nm with a peak at 311 nm. Subsequently, an 80 mJ/ cm^2^ UV dosage was applied two times per week for 8 months or until the tumours reached a maximum size of 1.5-2.5 cm.

To induce secondary tumours, immunodeficient NMRI female mice were subjected to a subcutaneous transplant into an area that overlaid the front/hind flank with equal number of flow-cytometry sorted cells (1 × 10^3^ cells per site) in a volume of 100 µl phosphate-buffered saline (PBS, pH 7.2). Tumours were observed and measured every week. Mice were sacrificed when tumours reached 1.0-1.2 cm, or a maximum of 10 weeks post-transplantation.

### BrdU (5-bromo-2ʹdeoxyuridine) labelling and tumour preparations

Tumour cells were labelled by injecting animals once intraperitoneally with 80 mg/g body weight BrdU (Invitrogen, USA). The mice were sacrificed 2 h, 10 days or 30 days post-injection. Tumours were isolated and fixed in 10% formalin solution or quickly frozen in O.C.T compound (Tissue-TEC, USA) on dry ice.

### Flow cytometry sorting

Tumour tissues were cut and digested by incubation with 0.25% trypsin for 20 min. Viable cells were examined using 0.4% trypan blue. Cells were kept in 10% Dulbecco's Modified Eagle Medium (DMEM) with 10% foetal bovine serum (FBS) filtered with a 0.45 µm filter. The cells were subsequently stained with phycoerythrin (PE)-conjugated rat anti-mouse α6-integrin antibody (1:50, BD Bioscience, USA), FITC-conjugated rat anti-mouse CD34 antibody (1:30, BD Bioscience, USA), or IgG control anti-mouse antibody for 45 min in the dark at 4°C. After washing, the appropriate amount of cell preparations were sorted based on the α6-integrin and CD34 expression status using a FACS Aria cell sorter (BD Bioscience, USA). The live cell population gate was estimated using forward and side scatter positioning, which was further confirmed by propidium iodide (PI) staining. A 488 nm laser was used to detect fluorescein isothiocyanate (FITC) with a 530/30 filter and a 532 nm laser for PE with 575/25 filter.

### Immunofluorescence staining

Immunostaining was performed with frozen tissue sections as previously described. Tissues were fixed in methanol at -20°C for 20 min. After blocking with PBS with 3% FBS, tissues were incubated with anti-BrdU antibody (1:50, abcam, USA) and/or anti-CD34 antibody (1:10, abcam, USA) for 60 min at room temperature. Fluorescently labelled anti-rabbit (1:200, abcam, USA) or anti-goat antibody (1:200, abcam, USA) was incubated with the tissues for 45 min. After washing four times with PBS, the slides were mounted with Mountshield that contained with DAPI. Fluorescent images were captured and processed using the Olympus BX41 microscopic imaging system (Center Valley, PA, USA).

### Colony formation assay

NIH3T3 (1 × 10^6^ cells) cells were freshly seeded onto 60 mm dishes in DMEM supplemented with 10% FBS for 2 days. To prevent NIH3T3 cell proliferation, mitomycin was used to treat the cells before seeding the tumour cells. Approximately 1 × 10^2^ flow-cytometry-sorted tumour cells were seeded into each well. After 10-14 days of culturing, dead cells were washed away, and clusters of cells were imaged and counted under a microscope. For the soft agar assay, 1 × 10^2^ tumour cells (isolated from animals) were plated in each well with 4 ml 0.4% agarose (SeaPlaque, BioProducts, Rockland, ME, USA) in DMEM supplemented with 10% FBS. The cells were grown for 3 weeks at 37°C with 5% CO_2_, and the colonies were stained and counted under the microscope.

### Statistical analysis

Statistical analysis was performed using Student's *t*-test or analysis of variance (ANOVA) followed by Dunnett's *t*-test. Results are presented as mean ± standard error of the mean (SEM). For all tests, *P* < 0.05 was accepted as statistically significant.

## Results

### UV-induced skin cancer contained slow-proliferative cancer-initiating cells

**Figure 1:**
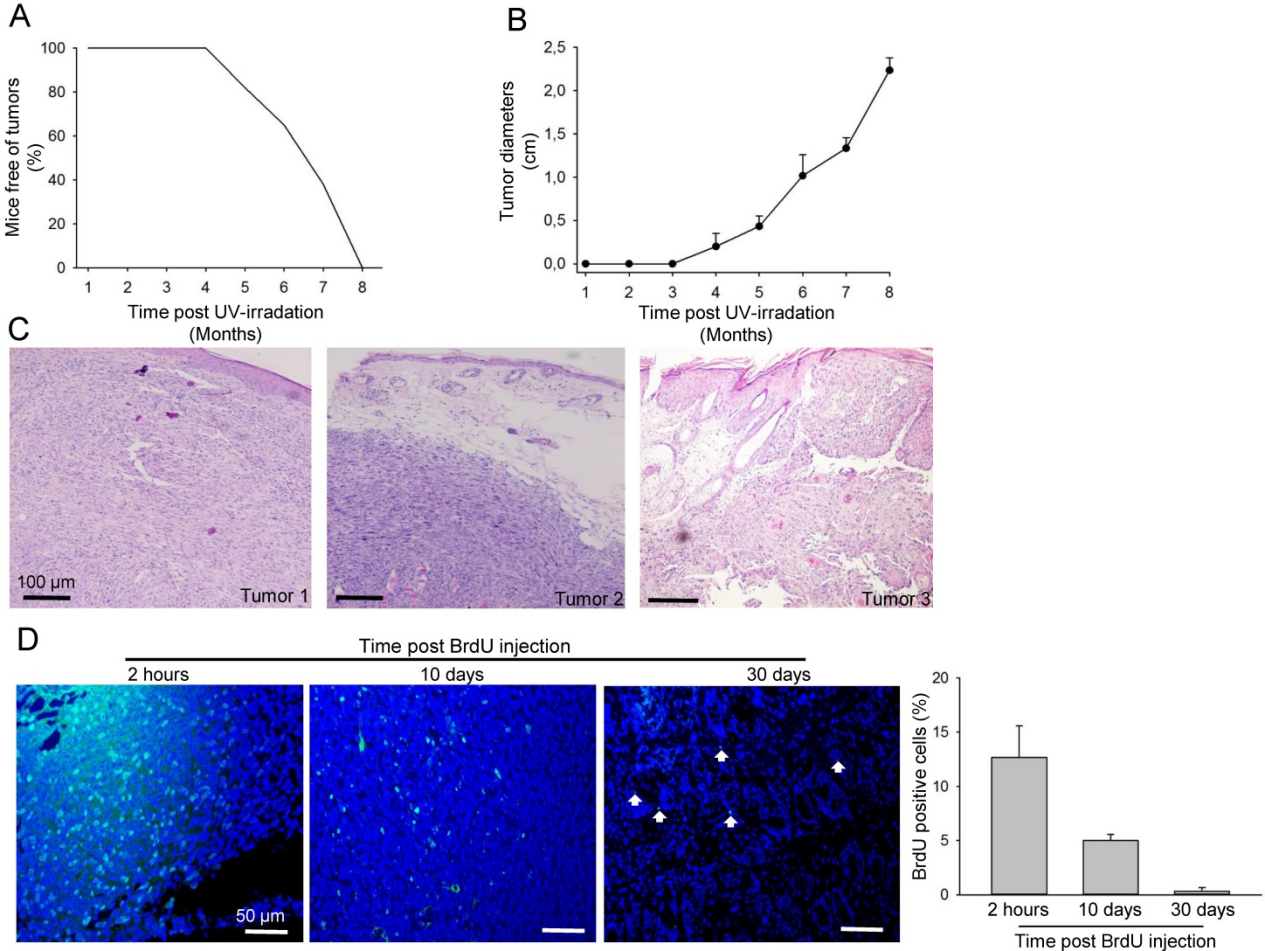
** Generation of UV-induced SCC model in mice. A.** Skin tumour occurrence in mice after UV-B irradiation over a period of 8 months (n = 28). **B.** Tumour growth, as measured by tumour diameter, in mice treated with UV-B irradiation over a period of 8 months. **C.** Haematoxylin-eosin-stained representative images of tumours on the back of mice exposed to UV radiation. **D.** Immunofluorescence staining with BrdU (green) antibody in tumour tissue biopsied 2 h, 10 days and 30 days post-BrdU injection. Percentages of BrdU-positive cells in the tumour tissue (n = 3 for each time point) are shown in the right panel.

**Figure 2:**
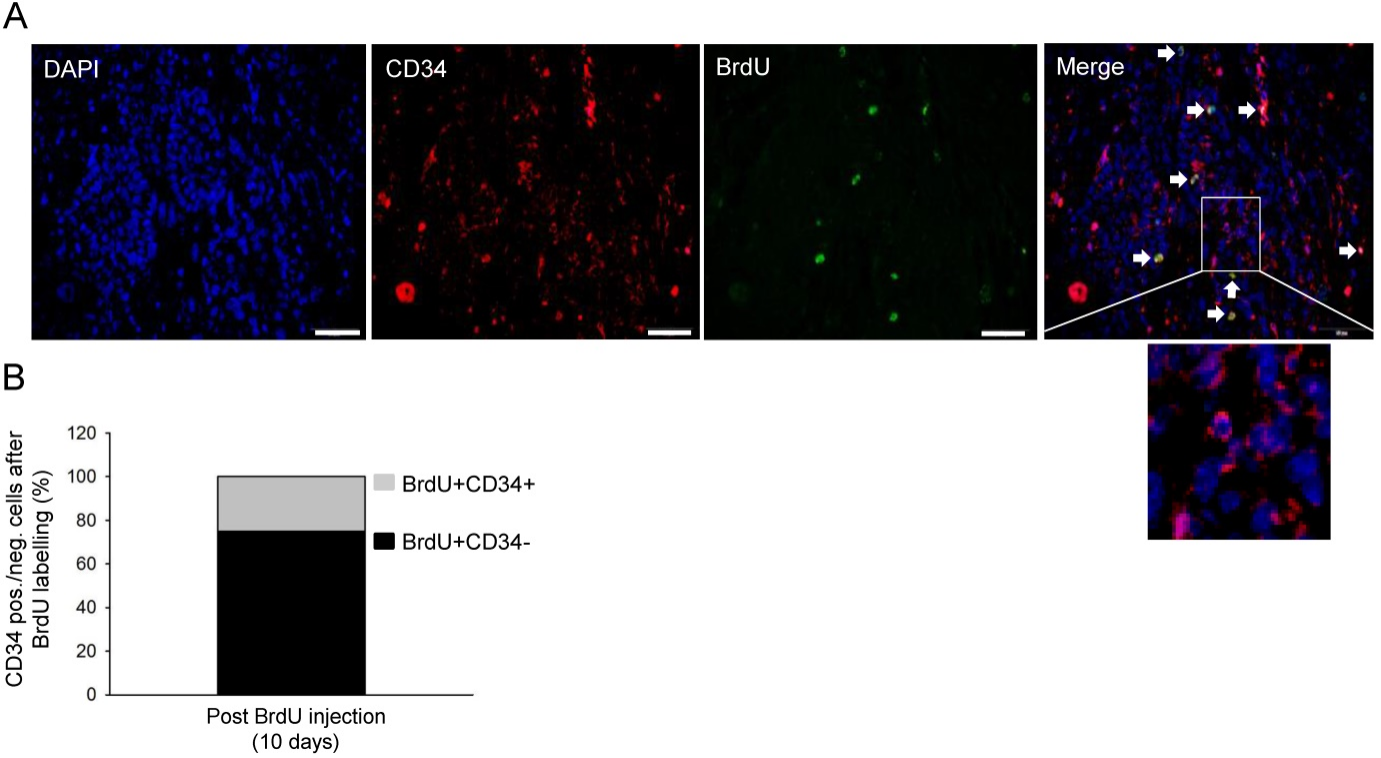
** UV-induced skin cancer contained slow-proliferative cancer-initiating cells. A.** Immunofluorescence staining for BrdU (green), CD34 (red), DAPI (blue), and Merge (higher magnification) in tumour tissue biopsied 10 days post-BrdU injection. Arrow heads show CD34 and BrdU positive cells. **B.** Percentages of CD34 positive/negative cells among BrdU-positive cells in tumour tissue biopsied 10 days post-injection.

To generate a UV-B induced skin cancer model, FVB mice were irradiated for up to 30 weeks. Three mice developed small tumours as early as 4.5 months after radiation, and all mice developed SCC after 8 months irradiation (Fig. 1A-B). Most of the SCC mice appeared to have a skin ulcer (Fig. 1C). In all cases, one tumour per mouse grew at the location exposed to UV irradiation; the animals were challenged with UV exposure until the tumour reached a maximum diameter of 1.5-2.5 cm (Fig. 1B). Haematoxylin and eosin staining confirmed the development of tumours including well differentiated and undifferentiated SCC (Fig. 1C). Long-term retaining cells (LRCs) in cancer tissues are recognised as cancer stem-like cells. To investigate the number of LRCs in the tumour, animals that were treated with UV-B for 8 months and developed SCC were injected with BrdU. The mice were sacrificed and tumours were further isolated after either 2 h, 10 days, or 30 days post-BrdU injection (Fig. 1D). The number of BrdU-positive cells was notably reduced from 2 h (12.1 ± 4.2%) to 10 days (4.9 ± 1.0%) to 30 days (0.30 ± 1%) after labelling (Fig. 1D). To further characterise the slow cycling tumour cells, tissue from tumours 10 days post-BrdU injection were stained for CD34 and BrdU. Approximately 80% of the BrdU-positive cells were indeed positive for CD34 (Fig. 2A-B and supplementary Fig. 1A). These results indicate that only a small fraction of tumour cells were slow-proliferative cells, which are defined as cells that retain BrdU 10 days or longer after BrdU injection. Most of the cancer cells proliferate quickly and lose the BrdU during the oncogenic processes.

### CD34^high^α6-integrin^high^ cells exhibited cancer-initiating cell properties and induced secondary tumours

**Figure 3:**
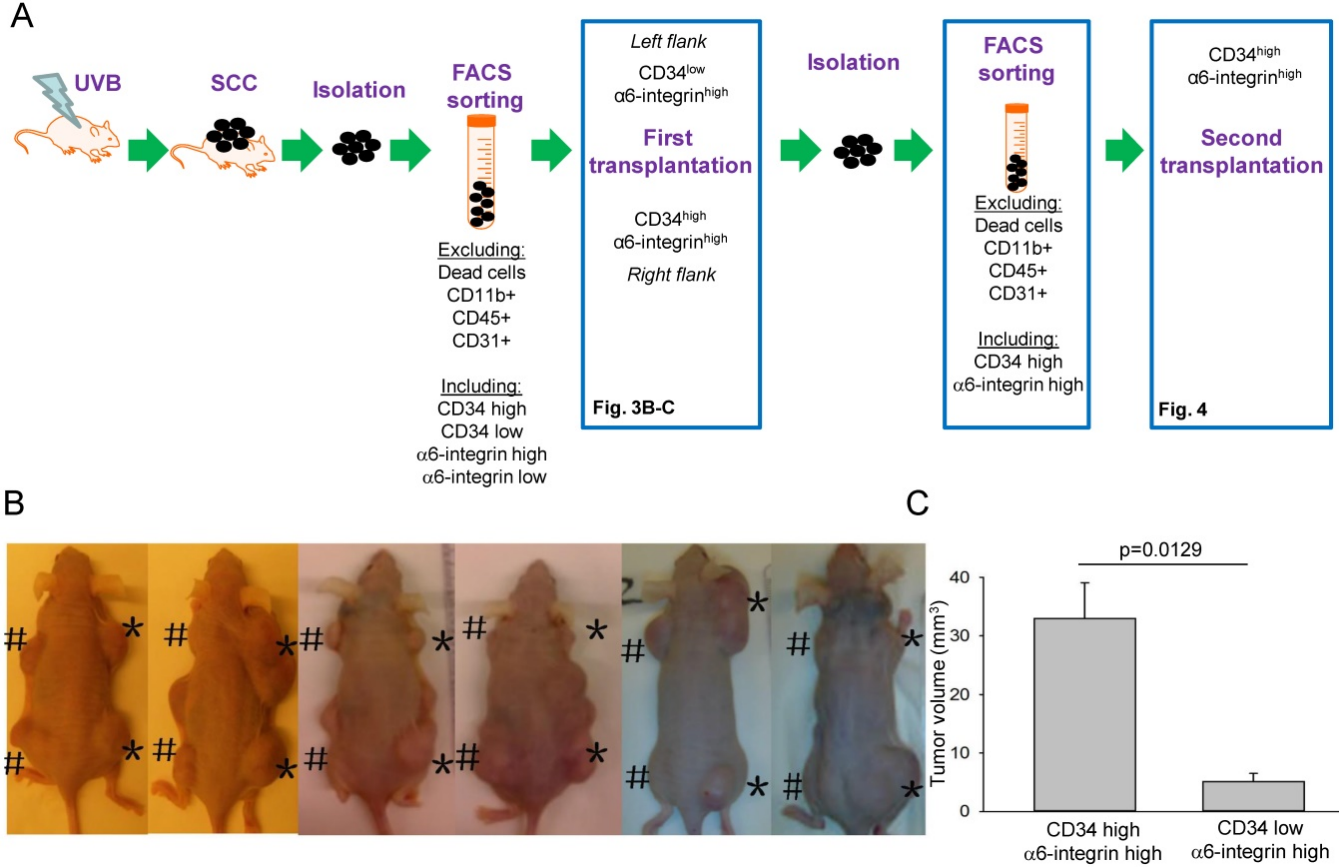
** CD34^high^α6-integrin^high^ cells displayed the properties of cancer-initiating cells. A.** Strategy to isolate CD34^low^α6-integrin^high^ and CD34^high^α6-integrin^high^ cells from SCC in animals and transplantation into immunocompromised animals. **B-C.** Representative images 10 weeks post-transplantation (subcutaneous administration) of CD34^low^α6-integrin^high^ cells on the left (#) and CD34^high^α6-integrin^high^ cells on the right (*) side of the same immunocompromised animal (B). CD34^high^α6-integrin^high^ showed a stronger tumour formation ability *in vivo* compared to CD34^low^ α6-integrin^high^ cells (C, n = 12).

**Figure 4:**
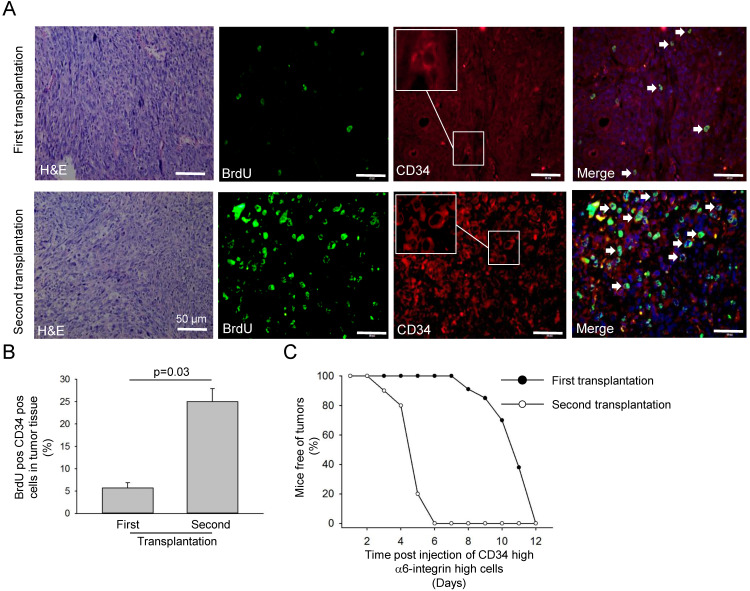
** Proliferating CD34-positive cells were increased in transplanted tumours. A.** Haematoxylin-eosin and immunofluorescence staining with anti-CD34 (red, including higher magnification) and anti-BrdU (green) antibodies of tumour tissues isolated from CD34^high^α6-integrin^high^ cells after first and second transplantation into immunocompromised animals. Arrow heads show CD34 and BrdU positive cells. **B.** Contribution of BrdU- and CD34-positive cells in tumour tissues isolated from CD34^high^α6-integrin^high^ cells after first and second transplantation into immunocompromised animals. Data represent the average of three independent experiments (n = 3). **C.** Tumour occurrence of CD34^high^α6-integrin^high^ cells after first and second transplantation into immunocompromised animals.

Flow cytometry analysis revealed that approximately 35.2 ± 2.3% of tumour cells were CD34 and α6-integrin positive, while 31.7 ± 1.9% of the cells were CD34 positive and α6-integrin negative. To investigate whether CD34 expression contributes to the cancer-initiating status of the α6-integrin-positive cells in UV-induced skin tumours, cells from SCC tumours were stained with PI to exclude the dead cells, lymphocytes (CD11b+), immune cells (CD45+) and endothelial cells (CD31+) in the tumour cell population. The remaining epithelial tumour cells were further separated into CD34^high^α6-integrin^high^ or CD34^low^α6-integrin^high^ populations (Fig. 3A, supplementary Fig. 1B and Supplementary Fig. 2). The same immunodeficient animal was transplanted with CD34^high^ α6-integrin^high^ (right side, marked as '*' Fig. 3B) or CD34^low^α6-integrin^high^ (left side, marked as '#', Fig. 3B). We observed that CD34-positive cells generated tumours more effectively and the tumours grew faster compared to CD34-negative cells (Fig. 3B-C). We also compared the number of CD34-positive cells that were labelled with BrdU in the original UV-induced SCC with those isolated from transplanted mice by co-immunostaining for CD34 and BrdU. The total BrdU-positive rate increased 4-fold in CD34-positive cells in transplanted tumours compared to original tumours (Fig. 4A-B). This result suggests that the CD34-positive cells in the transplanted tumour were significantly more proliferative compared to the CD34-positive cells within the original tumour tissue. To monitor whether CD34^high^ α6-integrin^high^ are cancer-initiating cells, we repeated the transplantation into immunocompromised mice and compared tumour development between the second and first transplantations. By day 6, almost all the mice transplanted second time with the CD34^high^α6-integrin^high^ cells developed tumours in comparison to day 12 observed after the first transplantations. (Fig. 4C). These data suggest that CD34^high^ α6-integrin^high^ cells acted as cancer-initiating cells in UV-induced SCC.

### CD34^high^ α6-integrin^high^ transplanted cells had a faster self-renewal ability

**Figure 5:**
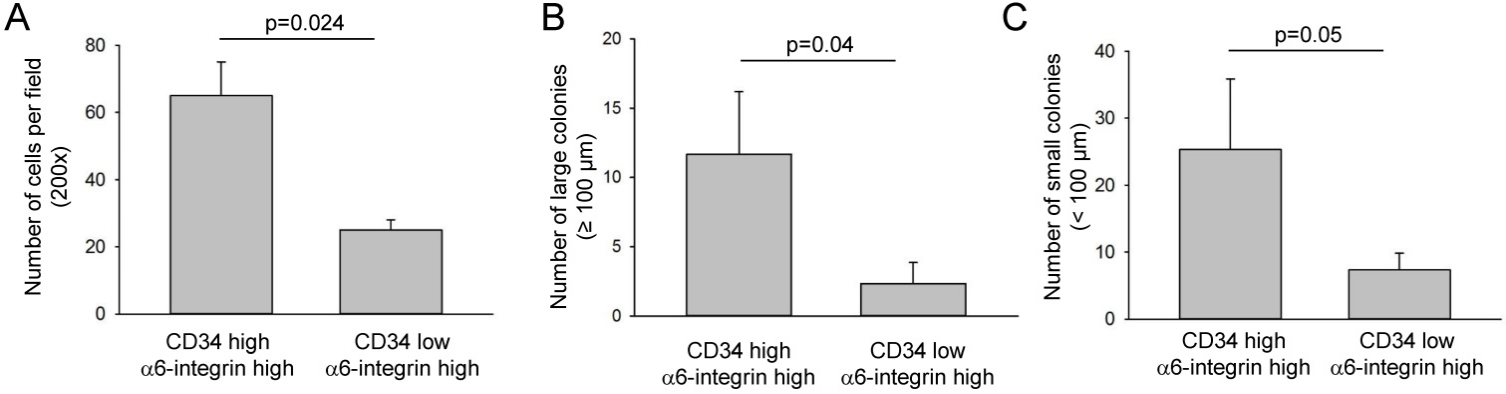
** CD34^high^α6-integrin^high^ cells displayed a stronger self-renewal ability *in vitro*. A.** CD34^high^α6-integrin^high^ or CD34^low^ α6-integrin^high^ cancer cells (shown in Fig. 2B) isolated from the first transplantation were grown on the top of mitomycin-treated NIH3T3 cells. After 14 days, the number of clones was counted and is presented as number of cells per field (n = 5). **B-C.** CD34^high^α6-integrin^high^ or CD34^low^α6-integrin^high^ cancer cells were cultured on a low-adherence surface using soft agar assay and phase contrast image displayed different colony sizes. Large clone size: ø > 100 µm, small clone: ø < 100 µm (n = 5).

To determine self-renewal ability and clonogenicity capabilities, CD34^high^α6-integrin^high^ and CD34^low^α6-integrin^high^ cancer cells isolated from the first transplantation were cultured on the top of mitomycin-treated NIH3T3 cells. After 14 days, the NIH3T3 cells were washed away and the numbers of clones was counted under a microscope. The number of clones formed by CD34^high^α6-integrin^high^ cells was significantly higher compared with CD34^low^α6-integrin^high^ cancer cells (Fig. 5A). The clonogenicity of CD34^high^ α6-integrin^high^ cells were also evaluated with a soft agar assay. CD34^high^α6-integrin^high^ cells exhibited elevated numbers and a larger clone size compared with CD34^low^α6-integrin^high^ cells (Fig. 5B-C).

## Discussion

SCC is the second most common form of skin cancer. It represents 20% of non-melanoma skin cancer cases in humans and is associated with substantial risk of metastasis. Approximately 90% of non-melanoma skin cancers are associated with exposure to ultraviolet (UV) radiation from the sun 25. This pathological situation can be mimicked in mice via chronic exposure to UV light. Lineage-tracing experiments have been used to evaluate the clonal growth of cancer initiating cells in skin squamous cell carcinomas 26, 27. Cancer initiating cells are usually a small population within tumours that can sustain long-term tumour growth, self-renewal, and promote induced secondary tumours after transplantation. A previous study that used a chemical carcinogenesis protocol (DMBA/TPA) demonstrated that SCC cells enriched for CD34 more effectively produce secondary tumours upon transplantation compared to the CD34-negative cells 18. Furthermore, CD34 null mice are resistant to SCC development using the DMBA-induced model 28. In addition to CD34, α6β1-integrin represents another marker for cancer-initiating cells. DMBA/TPA-induced SCC cells with high α6β1-integrin expression more effectively propagate in transplantation studies compared to low-α6β1-expressing cells 19. However, high α6β1-integrin expression promotes tumour-initiating abilities regardless of CD34 enrichment status.

In the present study, we examined whether cancer-initiating cells can be generated in UV-induced SCC in animals. UV exposure usually generates SCC within 4-7 months, which is longer than the time required for DMBA/TPA-induced tumours. Furthermore, UV-induced SCC tumours are usually easily transplantable and behave much more malignantly compared to chemical-induced skin cancer. The latter mostly generate benign papilloma. The majority of the slow-cycling BrdU-positive cells (i.e., BrdU positive 10 days post-injection) were CD34-positive cells. Previous research demonstrated that EdU is high in CD34^high^ tumour cells in genetically modified malignant tumours^19^. Consistently, our data indicated that in the UV-induced SCC, both CD34 and slow-cycling BrdU-positive cells only represented a minor fraction (approximately 5%) of the tumour cells.

Next, we investigated whether the CD34 status (CD34 high versus CD34 low) affects the propagation to the secondary tumour. We isolated CD34^high^α6-integrin^high^ cells; they propagated more effectively and grew more rapidly in transplantation experiments compared to CD34^low^α6-integrin^high^ cells. Additionally, this effect was confirmed because CD34^high^α6-integrin^high^ cells formed larger and increased number colonies compared to CD34^low^α6-integrin^high^ cells isolated from UV-mediated SCC. To find whether CD34^high^α6-integrin^high^ cells can accurately recapitulate and propagate *in vivo*, we used orthotropic xenograft mouse model. CD34-positive tumours isolated from the transplant harboured an increased number of slow-cycling BrdU-positive cells compared to primary tumour isolated from UV-induced animal. In addition, CD34 positive cells transplantation into immunocompromised mice generated secondary tumour after 31 days, whereas repeated transplantations into immunocompromised mice generated tumours in all animals after 16 days.

Epigenetic changes plays an important role in cancer initiating cells, there the component of poly-comb repressive complex 2 was required for epidermal cancer stem cell survival, migration, invasion, and tumour formation 29, 30. Micro RNA 23 was also shown to be downregulated by the oncogene leading to expansion of cancer stem cells in skin carcinogenesis model 31. The oncogenic Ras expression combined with p53 deletion in interfollicular epidermis/hair follicle lineages promotes development of different types of invasive SCC. These tumours were showing epithelial mesenchymal transition (EMT) features 11. In another recent study, tumours originating from hair follicle could undergo EMT compared to interfollicular epidermis originating tumours 32. In this aspect, chromatin and transcriptional profiling of these two different cell populations were the deciding factor in whether targeted onco-driven-cells developed into well-differentiated SCCs or more invasive tumours characterized by EMT 32. Furthermore, it needs to be determined whether CD34^high^α6-integrin^high^ cells are enriched with EMT-related gene signature.

Overall, our data suggest that expression of CD34^high^ compared to CD34^low^ isolated from UV-induced SCC could propagate effectively by exhibiting greater tumour initiating and self-renewal abilities. Future studies need to demonstrate whether additional cell surface markers such as β1 integrin and CD133 as well as skin cancer stem cell markers can enrich the population of the cancer initiating cells within CD34^high^α6-integrin^high^ in SCC that has been arising from UV-induction.

## Supplementary Material

Supplementary figures and tables.
