# Supplementary material for: High expression of CD34 and α6-integrin contributes to the cancer-initiating cell behaviour in ultraviolet-induced mouse skin squamous cell carcinoma
Source: J Cancer. 2020 Sep 30;11(23):6760–7. doi: 10.7150/jca.45819 (PMC7592010; doi:10.7150/jca.45819)
Supplement: Supplementary file 1 — Supplementary figures and tables. [file jcav11p6760s1.pdf]

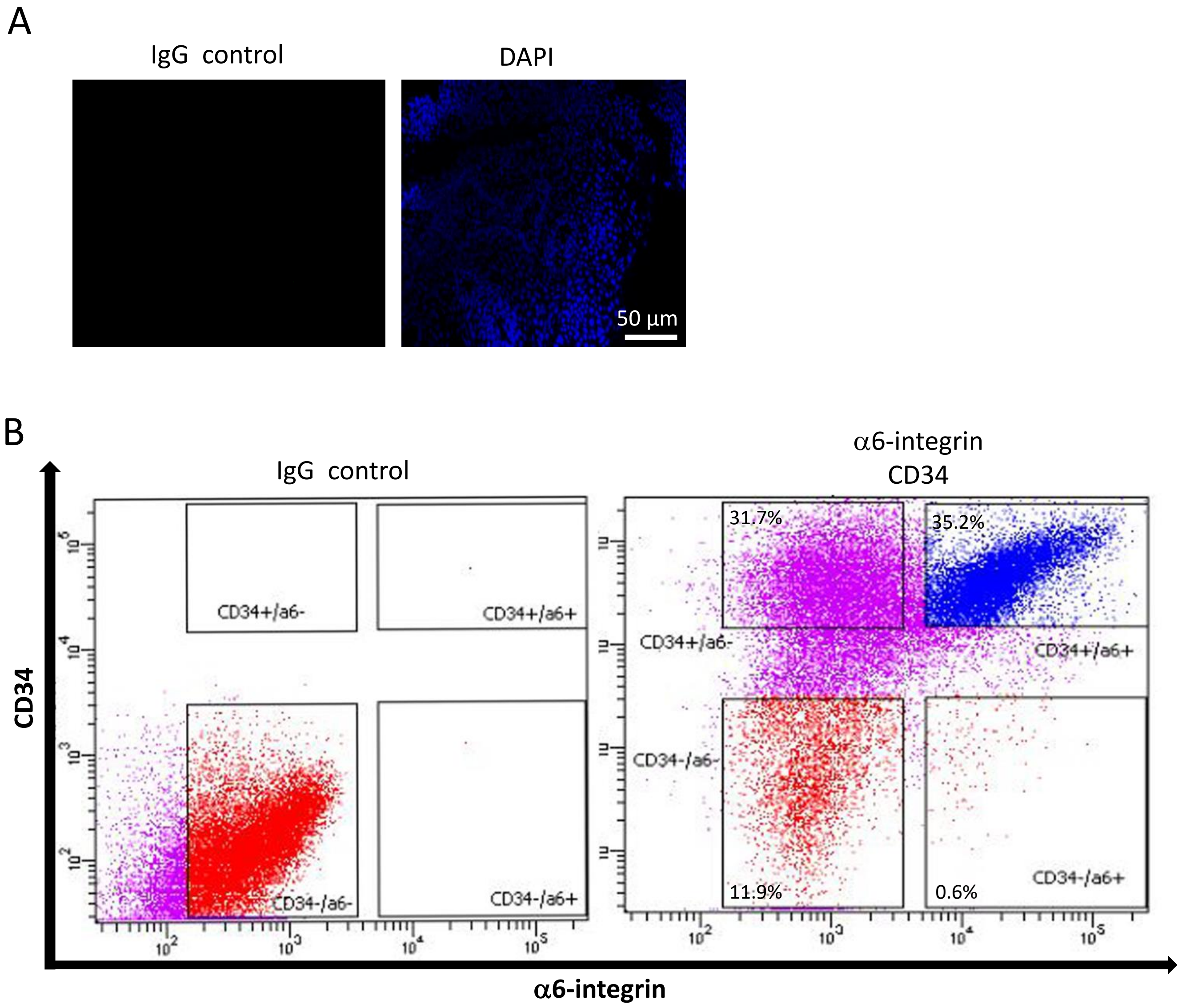

Supplementary Fig. 1

A. Immunofluorescence staining of UV-induced tumour tissue with IgG control (red) antibody and DAPI (blue).
B. Flow cytometry analysis of tumour cell population isolated from UV-treated animals stained with IgG control (left panel) or α6-integrin and CD34 antibodies in order to sort the cells.

Suppl. Fig. 1

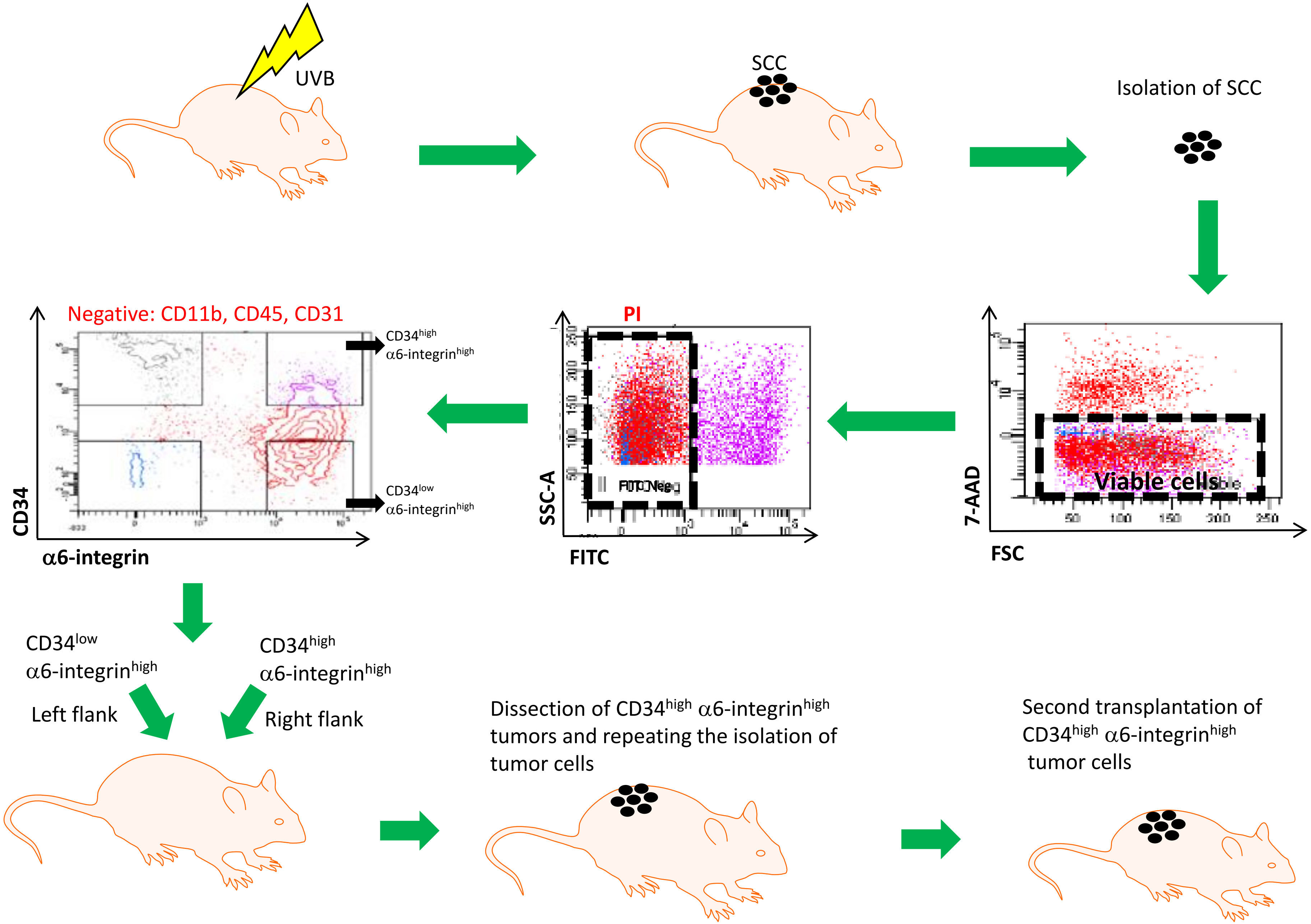

Supplementary Fig. 2

Strategy to isolate CD34^{low}α6-integrin^{high} and CD34^{high}α6-integrin^{high} cells from SCC in animals and transplantation into immunocompromised animals.

Suppl. Fig. 2
